# Topographic cues of a novel bilayered scaffold modulate dental pulp stem cells differentiation by regulating YAP signalling through cytoskeleton adjustments

**DOI:** 10.1111/cpr.12676

**Published:** 2019-08-19

**Authors:** Yu Du, Carolina Montoya, Santiago Orrego, Xi Wei, Junqi Ling, Peter I. Lelkes, Maobin Yang

**Affiliations:** ^1^ Guangdong Provincial Key Laboratory of Stomatology, Department of Operative Dentistry and Endodontics, Guanghua School of Stomatology, Affiliated Stomatological Hospital Sun Yat‐sen University Guangzhou Guangdong China; ^2^ Regenerative Health Research Laboratory Department of Endodontology, Kornberg School of Dentistry Temple University Philadelphia Pennsylvania; ^3^ Department of Oral Health Sciences, Kornberg School of Dentistry Temple University Philadelphia Pennsylvania; ^4^ Department of Bioengineering, College of Engineering Temple University Philadelphia Pennsylvania

## Abstract

**Objectives:**

Topographic cues can modulate morphology and differentiation of mesenchymal stem cells. This study aimed to determine how topographic cues of a novel bilayered poly (lactic‐co‐glycolic acid) (PLGA) scaffold affect osteogenic/odontogenic differentiation of dental pulp stem cells (DPSCs).

**Methods:**

The surface morphology of the scaffolds was visualized by scanning electron microscope, and the surface roughness was measured by profilometry. DPSCs were cultured on each side of the scaffolds. Cell morphology, expression of Yes‐associated protein (YAP) and osteogenic/odontogenic differentiation were analysed by immunohistochemistry, real‐time polymerase chain reaction, and Alizarin Red S staining. In addition, cytochalasin D (CytoD), an F‐actin disruptor, was used to examine the effects of F‐actin on intracellular YAP localisation. Verteporfin, a YAP transcriptional inhibitor, was used to explore the effects of YAP signalling on osteogenic/odontogenic differentiation of DPSCs.

**Results:**

The closed side of our scaffold showed smaller pores and less roughness than the open side. On the closed side, DPSCs exhibited enhanced F‐actin stress fibre alignment, larger spreading area, more elongated appearance, predominant nuclear YAP localization and spontaneous osteogenic differentiation. Inhibition of F‐actin alignments was correlated with nuclear YAP exclusion of DPSCs. Verteporfin restricted YAP localisation to the cytoplasm, down‐regulated expression of early osteogenic/odontogenic markers and inhibited mineralization of DPSCs cultures.

**Conclusions:**

The surface topographic cues changed F‐actin alignment and morphology of DPSCs, which in turn regulated YAP signalling to control osteogenic/odontogenic differentiation.

## 1 INTRODUCTION

The topography, microgeometry and mechanical properties, such as stiffness, roughness and extracellular forces, play significant roles in how scaffolds may regulate stem cells behaviour.^1^, ^2^, ^3^, ^4^ Biophysical properties of the scaffolds affect cell adhesion, migration, proliferation and differentiation.^5^ Cells perceive their microenvironment through mechanical cues, which can cause changes in cell morphology including changes in cytoskeleton arrangement.^6^ Amongst the three types of cytoskeletons (actin microfilaments, intermediate filaments and microtubules),^7^ actin presents as either a free monomer (G‐actin) or a linear polymer (F‐actin). The F‐actin cytoskeleton network plays a key role in regulating important physical processes, such as cell morphology, adhesion and proliferation.^8^, ^9^, ^10^ Specifically, differentiation of human mesenchymal stem cells (MSCs) into chondrocytes and osteoblasts is associated with structural changes of F‐actin networks.^11^, ^12^ F‐actin can activate several signalling molecules, which include the Rap1, Rho family GTPases, phosphoinositide 3‐kinase (PI3K) and YAP signalling.^9^, ^13^ The YAP/Tafazzin (TAZ) pathway reportedly works as a nuclear relay of mechanical signals.^9^ Phosphorylated YAP is automatically excluded from the nucleus, while non‐phosphorylated YAP translocates into the nucleus and actively regulates MSCs differentiation.^11^, ^14^, ^15^

Regenerative endodontics has been recognized as an alternative treatment modality for the permanent tooth with necrotic pulp and immature root. It is a biologically based procedure designed to physiologically replace the necrotic pulp and hence promote continued formation of root and apical closure. The ultimate goal is to regenerate a functional pulp‐dentin complex.^16^ Regenerative endodontics applies all the principles of regenerative medicine and tissue engineering, that is, it utilizes specific cells, three‐dimensional scaffolds and growth factors alone or in combination to regenerate new tissues.^17^, ^18^ The new protocol for regenerative endodontics has been introduced to clinics in 2004.^19^ In most cases, the regenerative endodontic treatment failed to regenerate pulp‐dentin tissue inside canals. In few cases, the ‘pulp‐like’ tissue was found inside the treated root canal, consisting of a mixture of dentin, bone, blood vessels, and some collagen components, but it lacked the spatial organization that is most characteristic for the pulp‐dentin complex.^20^, ^21^ To improve the outcomes, several biomaterials have been tested for their potential use as scaffolds in regenerative endodontics. One that has caught a lot of attention is platelet‐rich plasma (PRP). Studies have shown that using PRP adjunct to blood clot promotes root lengthening and thickening.^22^ Other materials, such as dentin matrix and peptide hydrogel (Puramatrix™), have also been tested in animal models and shown to have a certain level of regenerative potential.^23^ In addition, growth factors, such as TGFβ1, SDF‐1 and BMP, have been incorporated into scaffolds to promote stem cell differentiation and tissue regeneration.^24^ Although all these new scaffold materials support three‐dimensional cell cultures, their homogenous nature limits their capability to provide spatial control over cell activities, thus fail to provide the different zone for pulp (centre area) and dentin (peripheral area) regeneration.^25^

Recognizing these limitations, our laboratory has generated a novel, bilayered biomimetic tissue scaffold that is designed to provide spatial control of dentin and pulp tissue regeneration.^26^ The porous scaffold has two different layers, namely an open side and a closed side. On the open side, human dental pulp stem cells (DPSCs) penetrated into the scaffold through the channels, while on the closed side the cells rather proliferated on the surface and underwent spontaneous osteogenic differentiation. Importantly, the observed scaffold‐guided osteogenic differentiation on the closed side occurred in basal medium, in the absence of exogenous osteogenic inducers.^26^ We suppose this is likely due to the optimized topographic characteristic of our scaffold, which regulates the differentiation of DPSCs.^27^

The purpose of this study was to test our hypothesis that the open and closed sides of our scaffold provide different topographic cues, which change the cytoskeleton arrangement and target the YAP signalling pathway, leading to the spontaneous differentiation of DPSCs into osteoblasts/odontoblasts on the closed side.

## 2 MATERIALS AND METHODS

### 2.1 Scaffold fabrication and topographic testing

Membrane‐like PLGA (75:25; Evonik Industries) scaffolds were fabricated by diffusion‐induced phase separation (DIPS) technique as described previously.^26^ Briefly, 12% or 20% PLGA was dissolved in dimethyl sulfoxide (DMSO, Sigma) overnight. Then, the PLGA was cast on a glass plate and submerged into deionized water, independently. After changing the water five times, the PLGA membranes were carefully detached from the glass surface. The glass side of the 12% PLGA layer was referred as the ‘open side’, while the water side of the 20% PLGA layer was referred as the ‘closed side’. The two layers were finally laminated under vacuum (0.2 Torr) at 4°C for 24 hours.

The morphology of the freshly assembled, dry scaffolds was examined by scanning electron microscopy (SEM, FEI Quanta 450FEG). The surface roughness of the scaffolds was measured using a contact profilometer (Surfcorder SE 1700, Kosaka Labs) with a 2 μm radius tip, a spread of 2.5 mm and a speed of 0.5 mm/s.

### 2.2 Dental pulp stem cells culture on the scaffolds

Human DPSCs were purchased from AllCells LLC. These cells were guaranteed by company through 10 population doublings, and they were positive for CD105, CD166, CD29, CD90 and CD73 and negative for CD34, CD45 and CD133. DPSCs were cultured in a basal medium composed with α‐MEM (Gibco), supplemented with 10% foetal bovine serum (FBS, HyClone), penicillin (100 U/mL, HyClone) and streptomycin (100 μg/mL, HyClone) and incubated in a humidified incubator of 5% CO_2_ at 37°C. The PLGA membranes were cut into squares (2.5 × 2.5 cm^2^ or 3.5 × 3.5 cm^2^) and placed into 12‐well or 6‐well plates (Corning), with either the open side or the closed side facing upward. DPSCs (passages 4~6) were seeded at a density of 5 × 10^3^ cells/cm^2^ in the following experiments. The medium was changed every 3 days.

### 2.3 F‐actin fibre analysis

At days 1, 7 and 14, the PLGA membranes with attached DPSCs on either the open side or the closed side were fixed in 3.7% paraformaldehyde (Thermo Fisher) at room temperature for 15 minutes. Then, the cells were permeabilized with 0.3% Triton X‐100 (ThermoFisher) for 1 hour. Afterwards, the microfilaments were labelled with Alexa Fluor 546 Phalloidin (ThermoFisher). Images were captured in a Fluoview FV1000 (Olympus) laser scanning confocal microscope (LSCM). Orientation of the F‐actin fibres with respect to the long axis of the cells was determined, as described in the literature.^28^ Briefly, from the five microscopic fields (upper, lower, left, right and centre), six cells were randomly chosen from each field; therefore, a total of 30 individual cells were chosen from each group. For the fibre analysis, a reference line was drawn through the long axis of the cell. Five fibres from each cell were selected to obtain the average fibre angels. ImageJ software (NIH, version 1.51) was used to measure the angle between the long axis of the cell and each fibre (value between 0 and 90°). Fibres with the angles less than 10° were considered aligned.

### 2.4 Cell morphology and YAP signalling analysis

The intercellular localization of YAP in DPSCs was analysed by immunohistochemistry assay. For F‐actin inhibition studies, the cells were cultured on the closed side of the scaffolds in basal medium supplemented with 1 μg/mL of CytoD (Sigma, C8273) for 24 hours. For YAP inhibition experiments, the cells were cultured on the closed side of the scaffolds in basal medium supplemented with 0.5 μg/mL of verteporfin (inhibitor of YAP, Sigma, 129 497 785) for 7 days. The medium was changed every 3 days. The fixed and permeabilized cells were incubated with a primary antibody (rabbit polyclonal anti‐YAP, CST, 4912) overnight at 4°C, then washed and incubated for 1 hour at room temperature with a secondary antibody (Alexa Flour 488, CST, 8878). Cells were washed and stained with phalloidin for F‐actin and 4′, 6‐diamidino‐2‐phenylindole (DAPI, CST, 8961) for DNA/nucleus.

ImageJ software was used to analyze single‐cell morphology and YAP expression in the CytoD experiment. At day 1, the area of spreading, cell shape index (CSI) and aspect ratio (AR) were automatically measured from 30 single cells for each condition. The CSI is calculated using the formula (4π × area)/(perimeter)^2^. A CSI value of 1.0 indicates a circle, while 0.0 indicates an elongated polygon. The AR is the ratio of the major axis to the minor axis. By superimposing F‐actin and DAPI images with YAP images, the ratio of YAP expression in the nucleus vs. cytoplasm was calculated as (nuclear YAP/nuclear area)/(cytosolic YAP/cytoplasm area).

### 2.5 Alizarin Red S Staining and RT‐PCR assay

DPSCs were cultured on the open side in basal medium and the closed side in basal medium with verteporfin (0.5 μg/mL), respectively. For the Alizarin Red S (ARS, Sigma Aldrich) staining, the scaffolds were fixed at day 14 and stained with 0.1% Alizarin Red S (ARS, Sigma Aldrich) at 37°C for 1 hour to evaluate the mineralization of DPSCs. For the RT‐PCR step, RNA of each sample was extracted using the RNeasy Mini kit (Qiagen) at day 7. Then, 0.5 μg RNA of each group was used for cDNA synthesis by RT^2^ Easy First Strand Kit (Qiagen). RT‐PCR was carried out using the SSOAdvanced Universal SYBR Green Supermix (BIORAD). The following human‐specific validated primers (Biorad) were used: collagen 1 (COL1A1, qHsaCED0043248), YAP (qHsaCED0037679), alkaline phosphatase (ALP, qHsaCID0010031) and runt‐related transcription factor 2 (RUNX2, qHsaCID0006726). All procedures were performed according to the manufacturer's protocols. The expression levels of the target genes were normalized to glyceraldehyde‐3‐phosphate dehydrogenase (GAPDH, qHsaCED0038674) and calculated using the ∆∆CT method.^29^

### 2.6 Statistical analysis

Each experiment except for the single‐cell analyses was repeated independently at least three times. Student's *t *test was used to compare two samples within the same group. The results were analysed using SPSS 23.0 (IBM) software. All values were expressed as mean ± standard error, and statistical significance was set at a *P* < 0.05.

## 3 RESULTS

### 3.1 Topographic cues regulate actin organization of DPSCs

**Figure 1:**
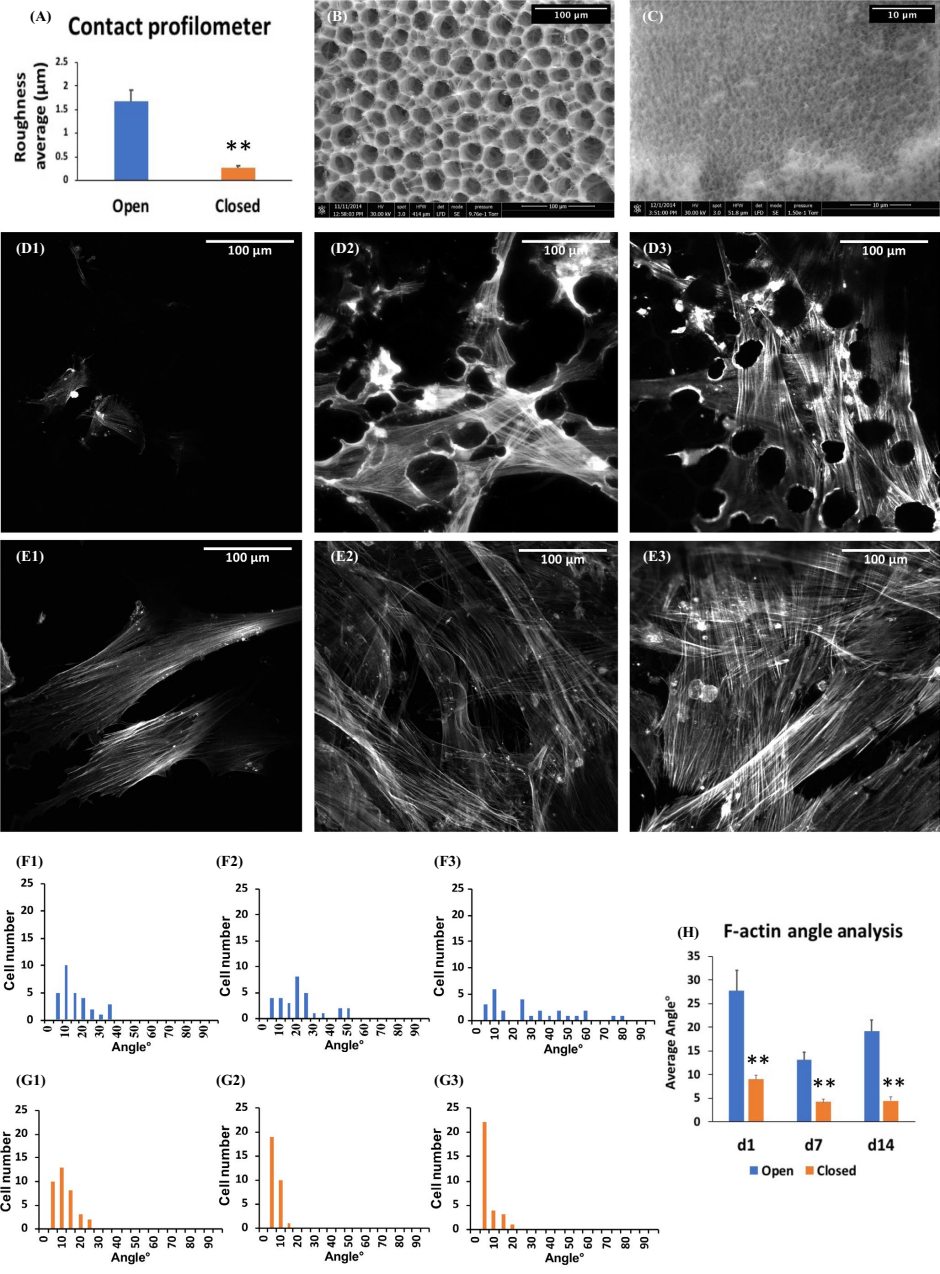
Topographic tests and F‐actin fibre analyses of DPSCs on each side of the bilayered scaffolds. A, The average roughness of the open side is higher than the closed side. B, SEM view of the surface of the open side. C, SEM view of the surface of the closed side. (D1~D3), The F‐actin arrangement of DPSCs on the open side at day 1, 7 and 14, respectively. (E1~E3), The F‐actin alignment of DPSCs on the closed side at day 1, 7 and 14, respectively. (F1~F3), The F‐actin to cell long axis angles on the open side showed a random distribution at day 1, 7 and 14, respectively. (G1~G3), F‐actin fibre alignments were higher on the closed side at day 1, 7 and 14, which also increased with time. H, DPSCs on the open side showed wider F‐actin fibre angles than those on the closed side. Scale bars of B, D and E: =100 μm, and scale bar of C: =10 μm. **indicated *P* < .01

The average roughness of the open side was 1.67 ± 0.25 μm, and that of the closed side was 0.27 ± 0.04 μm (Figure 1A). SEM revealed that the pores on the surface of the open side were significantly larger (~45 μm) than on the closed side (1 ~ 5 μm) (Figure 1B,C). These values are in good agreement with our previously published data.^26^

The F‐actin alignment of DPSCs was compared from day 1 to 14. Most of the cells exhibited random orientation on the open side (Figure 1D1~D3), while they showed elongated, spindle‐like morphologies on the closed side (Figures 1E1~E3). F‐actin alignment analysis demonstrated more cells with aligned fibres on the closed side than on the open side (Figures 1F1~F3,G1~G3). On the open sides DPSCs showed wider average fibre angles than those on the closed side (Figure 1H).

### 3.2 DPSCs exhibit different morphology and YAP localization on the different sides of the scaffold

At day 1, as compared to the cell morphology on the open side (Figures A1‐A5), DPSCs seemed to be more ‘stretched’ on the closed side (Figures B1‐B5). At day 7, DPSCs on the open side showed a randomly organized cytoskeleton, with both nuclear and cytosolic expression of YAP (Figures C1~C5). By contrast, DPSCs on the closed side showed parallel alignment and predominantly nuclear YAP localization (Figures D1~D5).

**Figure 2:**
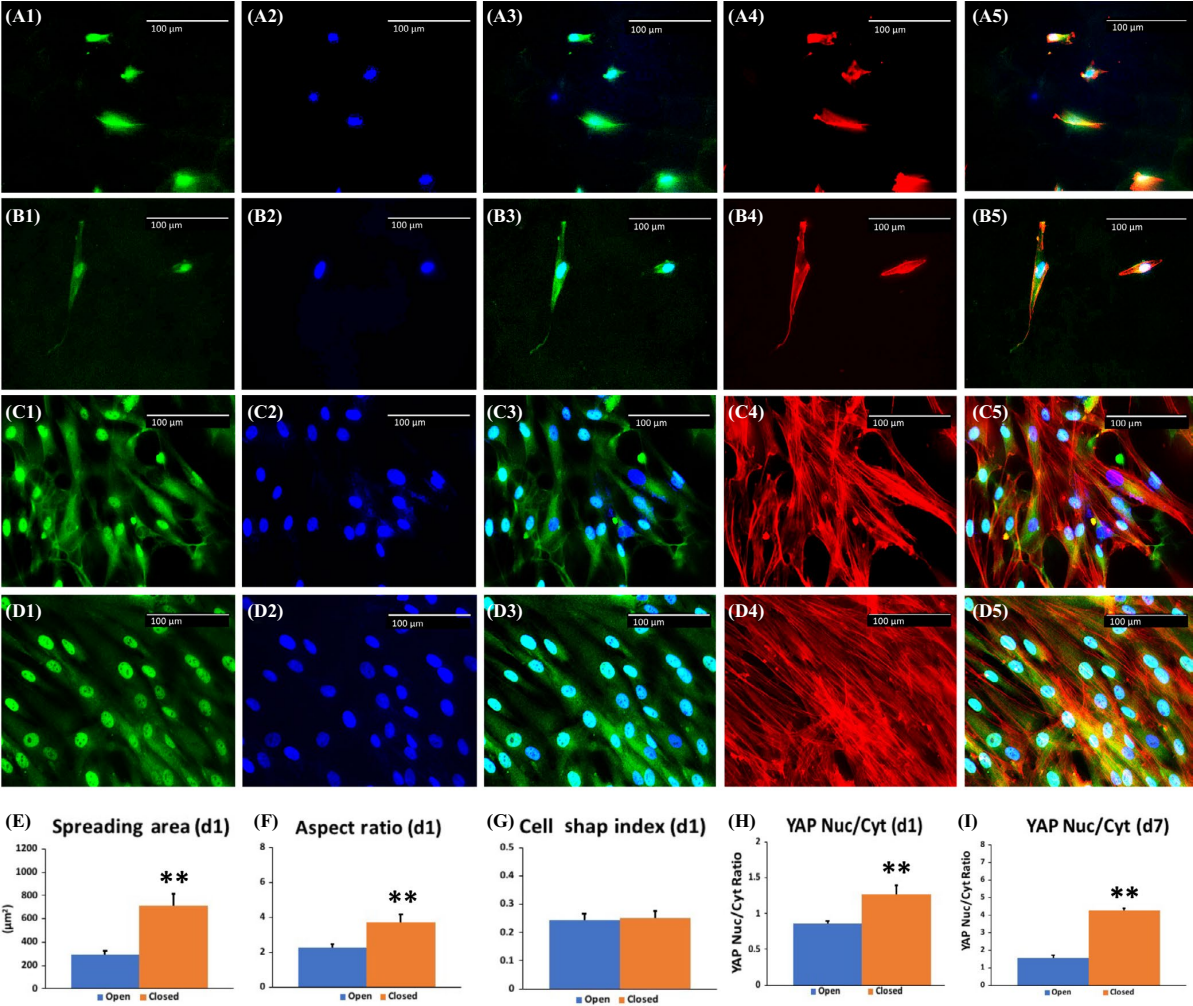
Intercellular YAP location and morphology analyses of DPSCs on the scaffolds. (A1~A5), DPSCs on the open side at day 1. (B1~B5), DPSCs on the closed side at day 1. (C1~C5), DPSCs on the open side at day 7. (D1~D5), DPSCs on the closed side at day 7. E, The average spreading area of DPSCs was significantly larger on the closed side. F, The average aspect ratio (AR) of DPSCs was significantly higher on the closed side. G, There were no statistical differences between cell shape indexes (CSI) of DPSCs on each side. H, The average YAP nuclear/cytosolic ratio in DPSCs was significantly higher on the closed side at day 1. I, The average YAP nuclear/cytosolic ratio in DPSCs was significantly higher on the closed side at day 7. Green fluorescence indicates YAP. Blue indicates DAPI. Red indicates F‐actin. A3, B3, C3 and D3 merged YAP and DAPI images. A5, B5, C5 and D5 merged all 3 channels. Scale bar = 100 μm. ** indicates *P* < .01

Further analysis of 30 single cells for each condition at day 1 indicated that the DPSCs had wider spreading area (545.2 ± 99.6 μm^2^) and higher AR (3.7 ± 0.5) on the closed side, while they had significantly smaller spreading area (295.2 ± 28.3 μm^2^) and lower AR (2.3 ± 0.2) on the open side (*P* < .01) (Figure 2E,F). However, there was no statistical difference between CSI of two sides (0.24 ± 0.02 vs 0.25 ± 0.02, *P* > .05) (Figure 2G). The YAP nuclear/cytosolic ratio was higher in DPSCs on the closed side on day 1 (Figure 2H). Consistently, DPSCs on the closed side on day 7 also exhibited a higher YAP nuclear/cytosolic ratio (Figure 2I).

### 3.3 F‐actin inhibitor changes cell morphology and YAP localization of DPSCs

**Figure 3:**
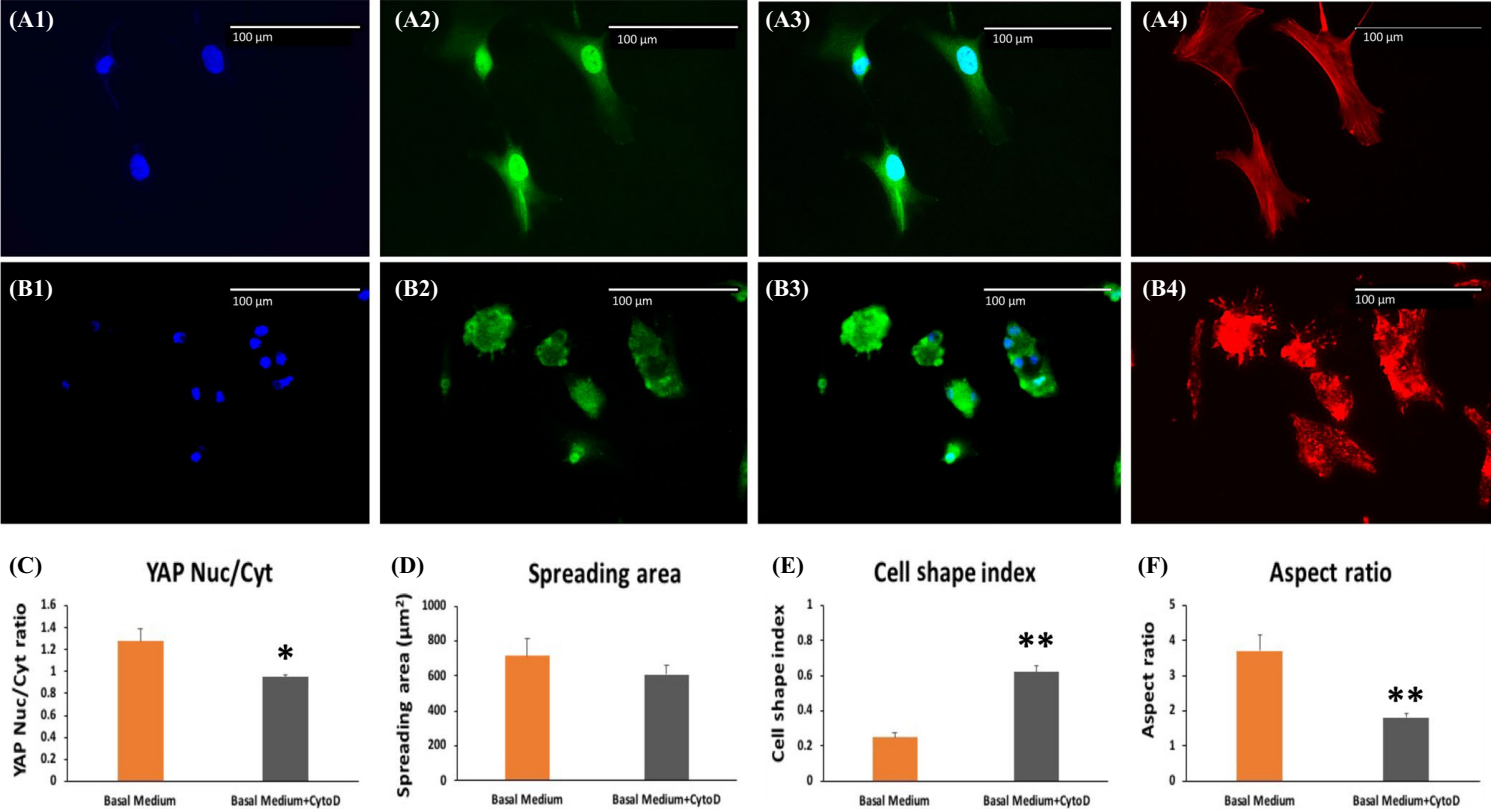
The effect of F‐actin inhibitor on nuclear YAP expression and morphologies of DPSCs on the closed side. (A1~A4), Representative images of DPSCs without CytoD treatment. (B1~B4), Representative images of DPSCs treated with CytoD for 24 h. C, YAP nuclear/cytosolic ratio was inhibited by CytoD for 24 h. D, The spreading areas showed no significant difference after CytoD treatment. E, The CSI was increased by CytoD treatment, which meant the cells' morphology changed to a more circle shape. F, The AR was decreased by CytoD, indicating that the number of spindle‐like cells declined. Green fluorescence indicates YAP. Blue indicates DAPI. Red indicates F‐actin. A3 and B3 are merged YAP and DAPI images. Scale bar = 100 μm. * indicated *P* < .05 and ** indicates *P* < .01

In the absence of cytochalasin D (CytoD), an established inhibitor of F‐actin polymerization, YAP was predominantly expressed in the nuclei of DPSCs cultured on the closed side (Figures A1~A4). Treatment with CytoD for 24 hours disrupted F‐actin organization and also led to YAP exclusion from the nuclei (Figures B1~B4). The YAP nuclear/cytosolic ratios were reduced by CytoD treatment (Figure 3C). Although the total spreading area was not statistically changed (Figure 3D), the CSI was increased (Figure 3E) and the AR (Figure 3F) was decreased, indicating that the cell morphology changed from spindle to circular shape.

### 3.4 Scaffold‐induced osteogenic differentiation of DPSCs on the closed side is attenuated by YAP inhibitor

**Figure 4:**
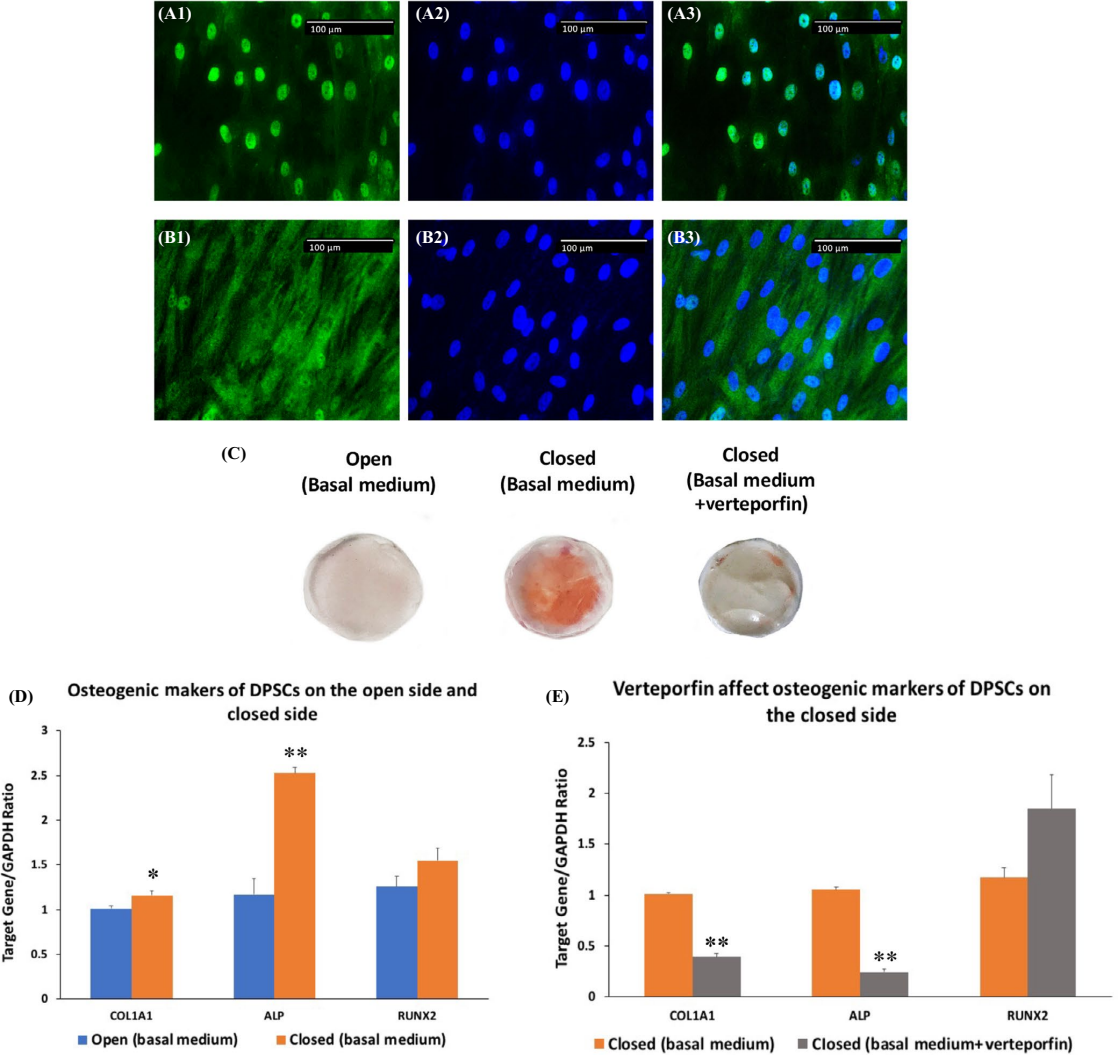
Enhanced osteogenesis of DPSCs on the closed side is attenuated by YAP transcriptional inhibitor. (A1~A3), Representative images of DPSCs without verteporfin treatment. (B1~B3), Representative images of DPSCs treated with verteporfin for 7 d. C, Representative ARS staining images of DPSCs cultured on the open side, the closed side and the closed side treated with verteporfin for 14 d. D, The mRNA levels of COL1A1, and ALP were elevated in DPSCs on the closed side. E, VP treatment down‐regulated the expression of COL1A1 and ALP of DPSCs on the closed side and showed no effect on RUNX2. Green fluorescence indicates YAP. Blue indicates DAPI. A3 and B3 are merged YAP and DAPI images. Scale bar = 100 μm. * indicates *P* < .05, and ** indicates *P* < .01

As shown previously, YAP was predominantly expressed in the nuclei of DPSCs cultured on the closed side of the scaffold (Figures A1~A3). After treatment with verteporfin for 7 days, nuclear YAP was essentially restricted to the cytoplasm (Figures B1~B3). In line with our previous findings (17), ARS staining showed that DPSCs cultured in basal medium on the closed side for 14 days underwent spontaneous osteogenic differentiation, which was not observed on the open side. The osteogenic differentiation was significantly diminished by treating the cells with the YAP inhibitor verteporfin (Figure 4C). After incubation in basal medium for 7 days, DPSCs showed an increase in the expression of ALP and COL1A1 on the closed side over that on the open side. No statistical significance was observed in the expression of RUNX2 between the two sides (Figure 4D). On the closed side, verteporfin treatment inhibited the expression of ALP and COL1A1 (Figure 4E).

## 4 DISCUSSION

As a main component of cytoskeleton, F‐actin actively responds to the surrounding environment. Once a cell senses physical cues from substrate attachment, the dynamic cytoskeleton network starts to reorganize accordingly.^30^ For instance, when endothelial cells were cultured on a grooved dimethylsiloxane substrate, F‐actin aligned along the grooves and ridges, but it did not show any preferential orientation on a smooth surface.^31^ Comparably, ridged or grooved silicon master surfaces also dramatically impacted alignment, elongation and aspect ratio of MSCs in a size‐ and shape‐dependent manner. MSCs on both planar and porous surfaces seemed to be more rounded when compared to their parallel orientation on ridged or grooved surface.^2^ We developed a bilayered PLGA scaffold in order to provide differential spatial guidance for DPSCs. SEM and profilometer results showed this scaffold had both larger pores and lower roughness on the open side than on the closed side, which may provide topographic cues to allow the penetration of DPSCs and subsequently affected cytoskeleton alignments, cell spreading areas and cell morphology. The cells on the closed side were ‘stretching’ with a remarkable degree of organization and directionality, while those on the open side were ‘crowding’. Although the underlying mechanism remains unknown, a recent study found that in responseto the fibronectin‐coated glass coverslips both the cell membrane roughness and focal adhesion size were increased the embryonic fibroblasts, via the enhancement of F‐actin polymerization.^32^

Cells in the dental pulp are intrinsically mechanosensitive to recognize and transduce mechanical changes into cellular responses.^33^ It is well established that the topographic cues of engineered tissue scaffolds can modulate dental stem cell behaviour. For example, increased surface wettability of a silk fibroin scaffold improved the attachment and proliferation of apical papilla stem cells.^34^ Decorated micropillars on polymeric substrate surfaces induced osteogenic differentiation of DPSCs.^35^ Specifically, the closed side of our scaffold promoted DPSCs osteogenic differentiation in the absence of osteogenic induction medium.^26^ We speculated that topographic cues, such as the lower roughness and smaller pore sizes on the closed side, affected DPSCs differentiation. The nanoscale surface roughness of the closed side (~260 nm) of our scaffold vs the micron scale surface roughness on the open side may play an important role in promoting DPSCs differentiation. This is consistent with other reports that a PLGA nanodiamond scaffold with a Ra at 0.349 μm promoted osteogenic differentiation of MSCs.^36^ Similarly, a layered PLGA membrane with a RA of ~0.3 μm facilitated osteogenic differentiation.^37^ In addition, the small pore size (1~5 μm) on the closed side seemed to be advantageous for DPSCs mineralization, while another study showed that porous PLGA microscaffolds with pore diameters of 10~30 μm supported the tri‐lineage differentiation potential of DPSCs.^38^

Growing evidence shows that cytoskeleton change is essential for the cell differentiation, although it may vary depending on differentiations towarding different cell lineages. When MSCs underwent osteogenic differentiation in vitro, actin fibres became disordered and robust after 2 weeks; while when they underwent chondrogenic differentiation, the cytoskeleton expanded in a parallel orientation originating from the centre of the cell.^8^ Others demonstrated that when MSCs were simulated by an osteogenic induction medium, the cytoskeleton modification was detected as an early event at day 1, which continued till day 14~21 to form abundant stress fibres and increased actin polymerization.^39^ Similarly, in the present study, the cells on the closed side of the scaffold displayed F‐actin polymerization at day 1, and the parallel alignment of F‐actin was observed through day 7~14, which was consistent with the spontaneous osteogenic/odontogenic differentiation of DPSCs without any addition of induction medium. Moreover, F‐actin may recruit different signalling molecules during the osteogenic differentiation of osteoblasts and MSCs. In the BMP2‐induced differentiation of osteoblastic precursor cell models, the RUNX2 gene was involved simultaneously with F‐actin reorganization.^40^ When MSCs were inducted by the osteogenic induction medium, p38 MAPK phosphorylation was required.^39^

The YAP/TAZ pathway is a transcriptional co‐activator of the Hippo pathway, which has been implied as a key mediator of mechanotransduction. When the Hippo pathway is activated, YAP and TAZ are phosphorylated and inhibited. When the Hippo pathway is inactivated, YAP and TAZ translocate into the nucleus, forming complexes with several transcriptional factors including TEAD, promoting transcription of target downstream genes.^41^ YAP signalling which is functionally required for MSCs differentiation, also plays a critical role during tooth development.^6^, ^42^, ^43^, ^44^ YAP signalling has been reported to promote osteogenesis and suppress adipogenesis in osteoblast cells.^45^ A recent study claimed that a static magnetic field (SMF) could promote nuclear YAP expression and inhibit the phosphorylation of YAP in DPSCs; however, SMF‐induced mineralization was suppressed by YAP knock‐down.^46^

F‐actin dynamic is a critical regulator of YAP/TAZ signalling. Dupont et al^6^ reported that a stiff matrix increased YAP/TAZ activity by increasing nuclear translocation; the same study also reported that cell spreading and organization of the actomyosin cytoskeleton activated YAP/TAZ. Driscoll et al^47^ reported that stretch increased the nuclear transfer of cytoskeletal strain and activated the YAP/TAZ pathway in MSCs. As seen in Figures 2 and 4, the level of nuclear YAP localization in DPSCs cultured for 7 days on the closed side of scaffold was significantly higher than that on the open side, which may contribute to the enhanced osteogenic differentiation observed on the closed side. In our hands, enhanced nuclear localization of YAP was consistent with higher F‐actin alignment, larger spreading area and higher AR in DPSCs. This finding is in line with previous studies.^6^, ^9^, ^47^

To further confirm the above findings, two distinct pharmacological inhibitors of F‐actin polymerization and YAP signalling were used. First, after CytoD treatment, DPSCs showed a dramatic decrease in nuclear YAP accumulation and an increase in cytosolic YAP expression, indicating that F‐actin polymerization regulates YAP translocation between cytoplasm and nucleus. Our result is consistent with previous studies demonstrating that CytoD treatment promoted production of intranuclear actin and led to nuclear export/exclusion of YAP.^48^ Previous study also showed that CytoD treatment decreased the cell volume and increased the cytosolic localization of YAP in MSCs cultured in hydrogels.^9^ Second, we treated DPSCs with verteporfin, a transcriptional inhibitor of YAP. Verteporfin was identified as an inhibitor of the interaction of YAP with TEAD and thus blocks transcriptional activation of YAP downstream genes.^49^ Although verteporfin treatment could remarkably up‐regulate cytoplasmic YAP in a series of cells, which may induce sequestration of cytosolic YAP through increasing levels of the cell‐cycle regulatory 14‐3‐3σ proteins, the studies related to osteogenic differentiation are very limited.^9^, ^50^ Verteporfin treatment in our experiment caused a significant down‐regulation of the expression of COL1A1 and ALP, as well as a reduced formation of mineralization nodules in DPSCs culture, but had no effect on RUNX2 expression. This is similar to a previous report that verteporfin dose dependently reduced mRNA levels of COL1A1, COL1A2, BSP and OCN, but not RUNX2 and osterix in osteoblast‐like cells.^51^

Taken together, our data suggest an important role of YAP signalling in the osteogenic/odontogenic differentiation of DSPCs. Our study demonstrates that YAP signalling is activated during DPSCs differentiation upon F‐actin alignment, regulated by topographic cues related to the roughness and pore sizes of our scaffold. We conclude that it is critical to understand dental stem cell behaviour on different scaffold surfaces and that cell behaviour can be manipulated by carefully controlling surface topographic properties. Our next step will investigate how this scaffold will guide the regeneration of pulp and dentin tissue inside root canals in vivo and whether YAP signalling is involved during the process of regeneration of the pulp‐dentin complex.

## CONFLICT OF INTERESTS

The authors deny any conflicts of interest related to this study.

## AUTHOR CONTRIBUTIONS

PL and MY designed the study; YD, XW and JL collected the data; XW, JL, CM and SO were involved in contribution of new reagents or analytical tools; YD, PL and MY analysed the data; YD, PL, CM, SO and MY prepared the manuscript.

## Data Availability

The data that support the findings of this study are available from the corresponding author upon reasonable request.
